# Preoperative cognitive training for the prevention of postoperative delirium and cognitive dysfunction: a systematic review and meta-analysis

**DOI:** 10.1186/s13741-024-00471-y

**Published:** 2024-11-30

**Authors:** Ka To Lau, Lok Ching Sandra Chiu, Janet Shuk Yan Fong, Albert Kam Ming Chan, Kwok Ming Ho, Anna Lee

**Affiliations:** 1 Faculty of Medicine, Chinese University of Hong Kong, Shatin, New Territories, Hong Kong, SAR China; 2 Department of Anaesthesia and Intensive Care, Faculty of Medicine, Chinese University of Hong Kong, Shatin, New Territories, Hong Kong, SAR China; 3 Department of Psychology, Faculty of Social Science, Chinese University of Hong Kong,, Shatin, New Territories, Hong Kong, SAR China; 4 Department of Anaesthesia, Pain and Perioperative Medicine, Prince of Wales Hospital, Shatin, New Territories, Hong Kong, SAR China

**Keywords:** Cognitive dysfunction, Cognitive rehabilitation, Confusion, Delayed neurocognitive recovery, Delirium, Neurocognitive disorders, Neuropsychological tests, Postoperative cognitive complications, Preoperative care

## Abstract

**Background:**

Postoperative delirium (POD) and postoperative cognitive dysfunction (POCD) are associated with major morbidity and mortality after surgery. This systematic review and meta-analysis determined whether preoperative cognitive training could reduce POD and POCD in patients undergoing elective surgery.

**Methods:**

Eligible randomized controlled trials were identified from CENTRAL, MEDLINE, EMBASE, Scopus, Web of Science, and CINAHL databases from inception to April 30, 2024. Two independent reviewers extracted data on trial characteristics and risk of bias for each trial. We rated the quality of reporting of cognitive training interventions using the template for intervention description and replication (TIDieR) and evaluated the overall certainty (quality) of evidence using The Grading of Recommendations, Assessment, Development and Evaluation (GRADE) system. Random-effects models were used to summarize the treatment effect of cognitive training. Post hoc trial sequential analyses (TSA) were performed for POD and POCD to differentiate between “no evidence of effect” and “evidence of no effect.”

**Results:**

Seven trials (four high risk and three unclear risk of bias) involving 864 participants (mean or median age between 66 and 73 years old) were considered eligible and subject to meta-analysis. The quality of reporting cognitive training interventions was fair to moderate. Most cognitive prehabilitation programs were home-based, unsupervised, computerized interventions requiring 2.3–10 h over 1–4 weeks before surgery. Cognitive prehabilitation did not reduce POD (risk ratio [RR] 0.82, 95% confidence interval [CI] 0.57–1.18; *I*^2^ = 30%; low certainty of evidence in five trials) or early POCD after surgery (RR 0.93, 95% CI 0.58–1.49; *I*^2^ = 67%; very low certainty of evidence in four trials) compared to usual care. Nonetheless, TSA suggested that the sample sizes were insufficient to exclude the effectiveness of preoperative cognitive training in reducing POD or POCD. The participants’ compliance rate was either not reported or mostly below 70%.

**Conclusions:**

Current evidence is insufficient to determine the beneficial effect of preoperative cognitive training on POD or POCD. Given the well-established benefits of long-term cognitive training on cognition in the elderly, the design of future cognitive prehabilitation trials should be adequately powered and incorporated with strategies to improve patient compliance.

**Supplementary Information:**

The online version contains supplementary material available at 10.1186/s13741-024-00471-y.

## Introduction

Postoperative delirium (POD) and postoperative cognitive dysfunction (POCD) are common after surgery, particularly among elderly patients. POD is a well-defined condition characterized by an acute onset of an altered consciousness and impaired attention following surgery (Krenk and Rasmussen 2011). By contrast, POCD is diagnosed through a series of neuropsychological tests that detect subtle cognitive declines, occurring between 30 days (delayed neurocognitive recovery) and 12 months (postoperative neurocognitive disorder) after surgery (Krenk and Rasmussen 2011; Evered et al. 2018).

The incidence of POD during hospitalization after major non-cardiac surgery is approximately 24%, while POCD affects about 47% of patients 1 month after surgery (Daiello et al. 2019). Among cardiac surgical patients, POD is also common, with an incidence ranging from 14 (Lee et al. 2017) to 46% (Saczynski et al. 2012). A decline in cognitive function scores, indicating POCD, after surgery varies from 49% at 1 month to 25% at 12 months (Saczynski et al. 2012). POD after surgery is linked to prolonged intensive care unit and hospital stays (Lee et al. 2018; Gleason et al. 2015; Brown et al. 2016), a lower likelihood of being discharged home (Gleason et al. 2015), a two-fold increase in hospital readmission within 30 days (Gleason et al. 2015), higher healthcare costs (Brown et al. 2016; Franco et al. 2001), and a seven-fold increase in long-term mortality (Moskowitz et al. 2017). POD is a strong predictor of POCD, suggesting that a hyperinflammatory process could be pathogenetically related to both conditions, despite their distinct presentations (Glumac et al. 2019, 2017).

Nonpharmacological multidisciplinary approaches are more effective than pharmacological interventions for managing POD (Igwe et al. 2020). Cognitive training to enhance neurological reserve and prevent POD and POCD (Vlisides et al. 2020), as part of multimodal prehabilitation, is an emerging field in perioperative medicine (Wong et al. 2022). The cumulative risks associated with multiple complex surgeries on cognition and neurodegeneration (particularly in the insula and superior temporal cortex) in the aging population underscore the importance of cognitive training in prehabilitation programs (Taylor et al. 2024). A systematic review of 97 studies in cognitively healthy and mildly impaired non-surgical adults over 60 years showed small improvements in cognitive functioning after cognitive training interventions (Hedges’ *g* = 0.30, 95% CI 0.25–0.35) (Mewborn et al. 2017). However, the effect of preoperative cognitive training on POD and POCD risk remains inconclusive, and no comprehensive systematic review has critically synthesized the evidence from recent randomized controlled trials (RCTs) (Greaves et al. 2023; Humeidan et al. 2021; Jiang et al. 2024; O'Gara et al. 2020; Ros-Nebot et al. 2024; Saleh et al. 2015; Vlisides et al. 2019).

We hypothesized that preoperative cognitive training might prevent POD and POCD. Our primary objective was to summarize the effect of preoperative cognitive training on POD and POCD risk in elective surgical patients. The secondary objectives were to evaluate the quality of existing studies and to identify areas for improvement in future research.

## Methods

A protocol for this systematic review was registered in PROSPERO (CRD42023435592, June 27, 2023). The conduct and reporting of this systematic review followed the guidelines from the Cochrane Handbook for Systematic Reviews of Interventions (Higgins et al. 2023) and the Preferred Reporting Items for Systematic Reviews and Meta-Analyses (PRISMA) (Page et al. 2021).

We identified eligible RCTs by searching the following electronic databases: Cochrane CENTRAL, OVID MEDLINE, OVID EMBASE, Scopus, Clarivate Analytics Web of Science, and CINAHL from inception to April 30, 2024 (Online Resource 1). We used the following text words “cognitive,” “mental,” “cognition,” “neurocognitive,” “brain,” “training,” “stimulation,” “therapy,” “exercise,” “game,” “intervention,” “programme,” “activity,” “CCT,” “postoperative delirium,” “POD,” “postoperative cognitive dysfunction,” “postoperative cognitive impairment,” “postoperative cognitive decline,” “postoperative cognitive disorder,” “postoperative neurocognitive dysfunction,” “postoperative neurocognitive impairment,” “postoperative neurocognitive decline,” “postoperative neurocognitive disorder,” “POCD,” and relevant MESH or subject headings in consultation with a medical librarian (Online Resource 1). There were no language restrictions for study inclusion for this review. We searched for ongoing clinical trials through the ClinicalTrials.gov and WHO International Clinical Trials Registry platform websites.

We included RCTs investigating the effects of cognitive training on POD and POCD in adult patients undergoing elective surgery. Cognitive training was defined as any program of regular activities comprising tasks designed to improve the participant’s cognitive abilities. We included all mediums of cognitive training administration, such as computerized training, written training, self-administered training regimens, and training by healthcare professionals. RCTs with other cognitive or non-cognitive interventions were only included if there was information comparing the isolated effects of cognitive training to the control group. We included RCTs that used reliable and validated batteries of neuropsychological tests designed to diagnose POD and POCD within the timeframe designated by each study as the outcome measures. These included, but are not limited to, the following tests: Confusion Assessment Method (CAM) (Inouye et al. 1990), CAM-ICU (Ely et al. 2001), Mini-Mental State Examination (MMSE) (Folstein et al. 1975), and the Montreal Cognitive Assessment (MoCA) (Nasreddine et al. 2005). The secondary outcome was the compliance rate of cognitive training, as defined by the authors, in each included study. Exclusion criteria were (a) case–control, case series, case reports, and cohort study designs; (b) children, critically ill adults, and adults undergoing emergency surgery; (c) multicomponent program or combined therapy where the effect of cognitive training could not be isolated; and (d) studies without baseline neuropsychological assessments before the cognitive training intervention. Five review authors independently, and in duplicate, screened article titles, abstracts, and full texts to identify eligible studies using Covidence, a web-based collaboration software platform (Covidence systematic review software 2023).

Data from each included RCT was independently extracted using a standardized form by two review authors using the Covidence software (Covidence systematic review software 2023). For each trial, we collected data on the title, authors, publication name, year of publication, publication language, funding, setting, eligibility criteria, number of participants randomized, age and sex of the study participants, type of elective surgery, cognitive training program characteristics, outcome measurements and timepoints, training compliance rate and risk of POD and POCD, or changes in postoperative cognitive decline. Any disagreements were resolved by consulting a third reviewer. Data was entered into Review Manager 5.4 (Copenhagen; The Nordic Cochrane Collaboration) by one author and verified by another author before conducting the meta-analyses.

Two review authors independently assessed the risk of bias for all included RCTs using the Cochrane Collaboration’s tool for assessing the risk of bias version 2.0 (Higgins et al. 2023) in the Covidence software (Covidence systematic review software 2023). The risk of bias levels for the seven domains (random sequence generation, randomization process, effect of assignment to interventions, effect of adherence to interventions, missing outcome data, measurement of outcome, selection of reported results) were classified as high, unclear, or low risk. A high-quality trial was defined as having all domains classified as having a low risk of bias. Similarly, a low-quality trial was defined as having one or more domains classified as having a high risk of bias. A third reviewer resolved any disagreements.

The Grading of Recommendations, Assessment, Development and Evaluation (GRADE) system was also used to evaluate the overall certainty (quality) of evidence by considering the following factors: study design, risk of bias, inconsistency, indirectness, imprecision, publication bias, and magnitude of effect (Guyatt et al. 2008). The level of evidence was classified as high, moderate, low, or very low certainty (Guyatt et al. 2008).

As the cognitive training intervention was relatively new and unstandardized in the prehabilitation setting, we assessed the quality of reporting of the interventions included in the RCTs based on the template for intervention description and replication (TIDieR) checklist and guide (Hoffmann et al. 2014). The TIDieR checklist contains 12 items: (1) a brief description of an intervention; (2) rationale, theory, and goal of the interventions; (3) descriptions of any physical/informational materials used; (4) procedures, activities, and processes used; (5) intervention provider; (6) modes of delivery; (7) location and facilities required; (8) dosage and duration of the interventions; (9) tailoring method (if any); (10) modification occurred (if any); (11) any planned measures to assess intervention adherence/fidelity; and (12) extent to which the intervention was delivered as planned so as to ensure that there were sufficient details for replicating research findings (Hoffmann et al. 2014). The quality of reporting using the TIDieR scoring method was also evaluated (Yamato et al. 2018). The summary score ranged from 0 (poor reporting) to 20 (good reporting); the tailoring and modification of intervention items were not applicable in the included RCTs and were therefore omitted in calculating the summary score (Yau et al. 2021).

For the dichotomous and continuous outcomes, we reported the risk ratio (RR) and mean difference (MD) values with 95% confidence intervals (95% CIs) as appropriate. The DerSimonian and Laird random-effects model was used as clinical and methodological heterogeneity among the included RCTs was expected. We explored the possible causes of high heterogeneity by performing a subgroup analysis by the type of surgery (noncardiac vs. cardiac). The statistical heterogeneity was assessed using the *I*^2^ statistic: an *I*^2^ value of < 30% was considered low, 30–60% was moderate heterogeneity, and 50–90% was substantial heterogeneity (Higgins et al. 2023, 2003). A sensitivity analysis was planned to confirm the robustness of our results by restricting the analysis to better-quality trials only, but there was no high-quality trial. Publication bias was assessed if there were 10 or more trials (Higgins et al. 2023). Data analysis and forest plots were performed and drawn using Review Manager 5.4 software (Copenhagen; The Nordic Cochrane Collaboration). All statistical tests were performed as two-sided tests with an alpha level of significance set at 0.05.

To differentiate between “no evidence of effect” and “evidence of no effect” from the meta-analysis results, a post hoc trial sequential analysis (TSA) (Wetterslev et al. 2017) was performed using the TSA software version 0.9.5.10 beta software, Copenhagen Trial Unit, Centre for Clinical Intervention Research, Copenhagen, Denmark (Thorlund et al. 2017). We estimated the required information size (sample size needed to achieve adequate power for one or more new trials to add to the meta-analysis to provide more firm evidence of the beneficial (if any) effect of cognitive training to prevent POD and POCD). The TSA approach adjusted for the trial sequential monitoring boundaries for both POD and POCD models with the following assumptions: type-1 error alpha of 5%, power at 80%, and relative risk reduction of 25% (control rates for POD and POCD set at 30% and 40%, respectively) with an adjustment for random-effects model-based variance heterogeneity.

## Results

### Search results and study characteristics

**Fig. 1:**
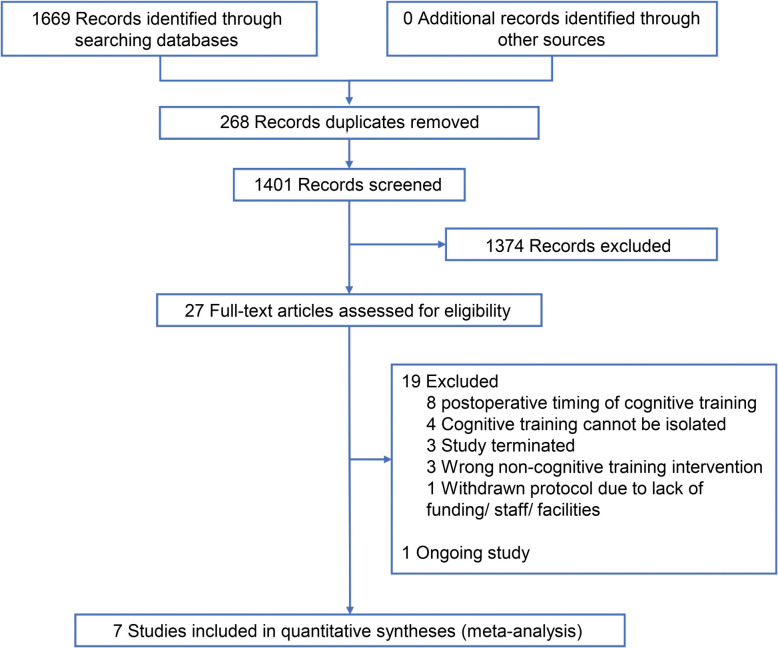
PRISMA flow diagram

Of the total 1669 records screened, seven trials (Greaves et al. 2023; Humeidan et al. 2021; Jiang et al. 2024; O'Gara et al. 2020; Ros-Nebot et al. 2024; Saleh et al. 2015; Vlisides et al. 2019) involving 864 participants were considered eligible and subject to meta-analysis (Fig. 1). There is one ongoing study with a protocol published (NCT04493996) (Butz et al. 2022). The excluded studies with reasons for their exclusion are described in Online Resource 2.

The included trials were conducted in the USA (Humeidan et al. 2021; O'Gara et al. 2020; Vlisides et al. 2019), Australia (Greaves et al. 2023), China (Jiang et al. 2024; Saleh et al. 2015), and Spain (Ros-Nebot et al. 2024), with participants who underwent cardiac surgery (Greaves et al. 2023; Jiang et al. 2024; O'Gara et al. 2020), non-cardiovascular and non-neurological surgery (Humeidan et al. 2021; Ros-Nebot et al. 2024; Vlisides et al. 2019), and gastrointestinal surgery (Saleh et al. 2015) (Table 1). The sample sizes ranged from 45 to 268 participants, and the mean or median age of participants in the included trials ranged from 66 to 73 years (Greaves et al. 2023; Jiang et al. 2024). The proportion of males varied between 35 and 83% (Greaves et al. 2023; Jiang et al. 2024).

**Table 1:** Characteristics of included trials

Author, year	Surgical population^a^	Cognitive training intervention	Comparator	Outcome measurement(s)
Greaves et al. 2023	45 CABG ± concomitant surgery	HappyNeuron Pro (targets psychomotor speed, attention, memory, executive function) Plan: 45–60 min every other day for 1–2 weeks; a total of 2.6 to 3 h Mode: Home-based, computerized	Usual care	POD daily till discharge; POCD change by NPT from the baseline to discharge, 4 and 6 months
Humeidan et al. 2021	268 noncardiac, non-neurological surgery	Lumosity (targets memory, process speed, attention, flexibility, problem-solving) Plan: 1 h daily for at least 8 days; a total of 10 h Mode: Home-based, computerized	Usual care	POD daily till discharge or till postoperative day 7
Jiang et al. 2024	218 CABG surgery	The Light of Future (targets memory, imagination, reasoning, reaction time, attention, processing speed) Plan: 20–30 min 2 or 3 times/day for at least 10 days; a total of 10 h Mode: Home-based, digital app	Usual care	POD daily till postoperative day 7; POCD change at discharge or postoperative day 7 and 1 month
O'Gara et al. 2020	45 cardiac surgery (CABG/ AVR)	Lumosity (targets memory, process speed, attention, flexibility, problem-solving) Plan: 15 min twice/day for at least 10 days; a total of 5 h Mode: Not specified	Usual care	POD daily till discharge or till postoperative day 7; POCD change at discharge, 1, 3, and 6 months
Ros-Nebot et al. 2024	80 noncardiac surgery	Sincrolab (targets memory, attention, executive function, problem-solving) Plan: 15 min daily for 10 days; a total of 2.5 h Mode: Home-based, digital app	Usual care	POCD change by NPT at postoperative days 7 and 30
Saleh et al. 2015	147 gastrointestinal tumor resection via laparotomy	Method of Loci (targets associative learning, memory) Plan: 60 min every other day for 1 to 4 weeks; a total of 3 h Mode: Hospital training	Usual care	POCD change by NPT at postoperative day 7
Vlisides et al. 2019	61 noncardiac, nonvascular, non-intracranial surgery	Brain HQ (targets executive function, attention, working memory, visuospatial processing) Plan: 20 min daily for at least 7 days; a total of 2.3 h Mode: Home-based, computerized	Usual care	POD twice daily from PACU till postoperative day 3; POCD change by NPT from the baseline to postoperative day 3

*AVR* Aortic valve replacement, *CABG* Coronary artery bypass graft, *NPT* Neuropsychological tests, *PACU* Postanaesthesia care unit, *POCD* Postoperative cognitive dysfunction, *POD* Postoperative delirium

^a^Number of patients randomized

The cognitive training modalities and plans used in the included trials were variable. Hospital-based cognitive training was administered by trained personnel in one study (Saleh et al. 2015), whilst the other six trials used computerized, home-based cognitive training programs on laptops, electronic tablets, or mobile phones (Greaves et al. 2023; Humeidan et al. 2021; Jiang et al. 2024; O'Gara et al. 2020; Ros-Nebot et al. 2024; Vlisides et al. 2019). Each training session varied between 15 and 60 min with a frequency ranging from thrice a day to every other day. The total recommended hours of cognitive prehabilitation ranged from 2.3 to 10 h, starting between 1 and 4 weeks before elective surgery. However, the actual training time achieved often deviated from the plan due to poor patient compliance.

### Methodological quality and reporting of included trials

**Fig. 2:**
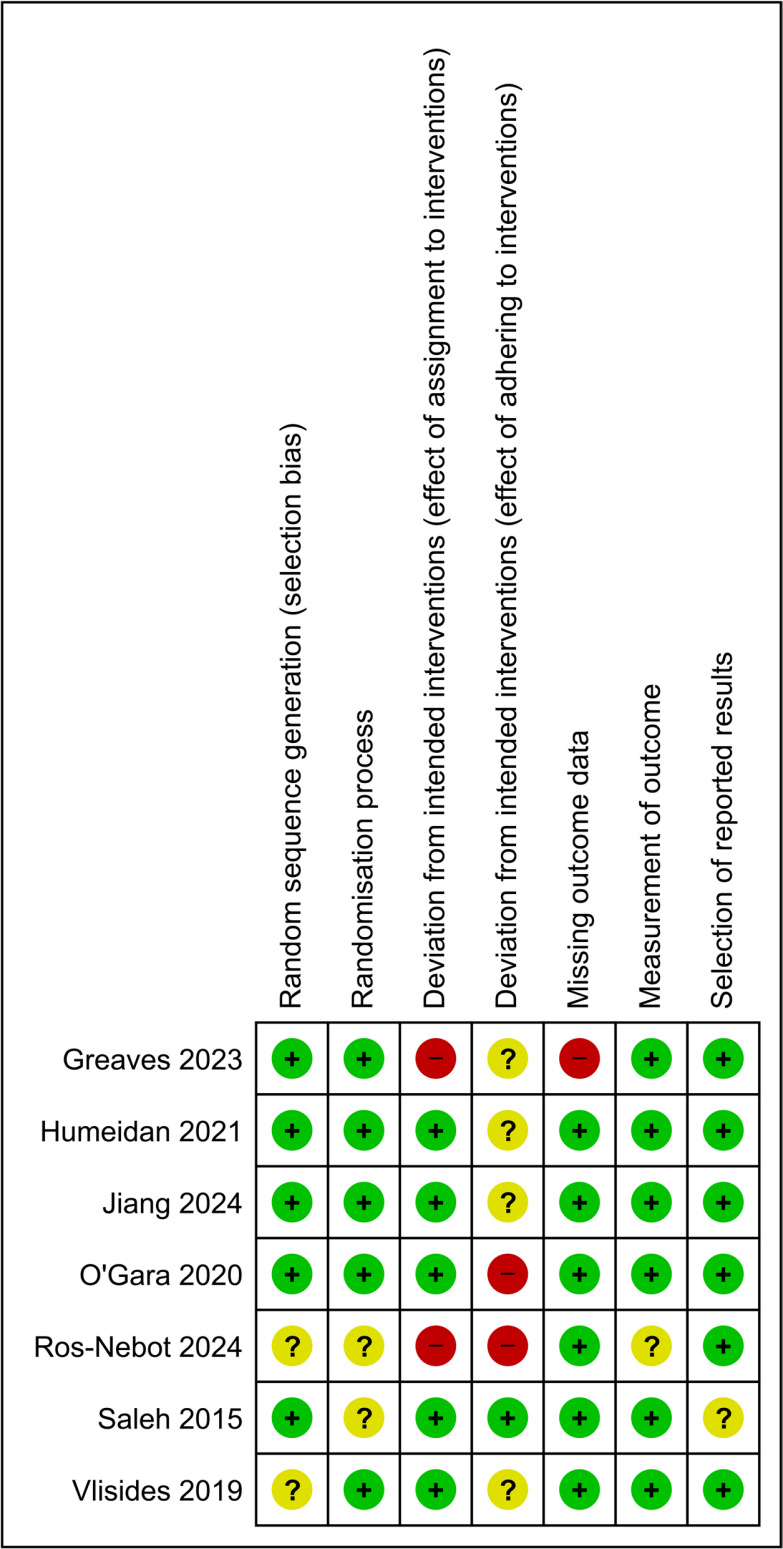
Risk of bias items for each trial

Of the seven trials, three (Greaves et al. 2023; O'Gara et al. 2020; Ros-Nebot et al. 2024) had at least one domain that was rated as high risk (Fig. 2). The trials conducted by Greaves and colleagues (Greaves et al. 2023), Ros-Nebot and colleagues (Ros-Nebot et al. 2024), and Saleh and colleagues (Saleh et al. 2015) used per-protocol analyses and only analyzed participants who met their adherence criteria. All other trials used the intention-to-treat analysis, including participants regardless of their compliance with the recommended hours of cognitive training.

The results of the TIDieR evaluation of the quality and completeness of the cognitive training intervention reporting are shown in Online Resource 3. The overall quality of the intervention reporting was rated as fair to moderate. Common insufficient details were in what information materials (with links) were used, who (qualifications and expertise) provided the intervention, and the exact locations where the cognitive training sessions took place.

### Postoperative delirium

**Fig. 3:**
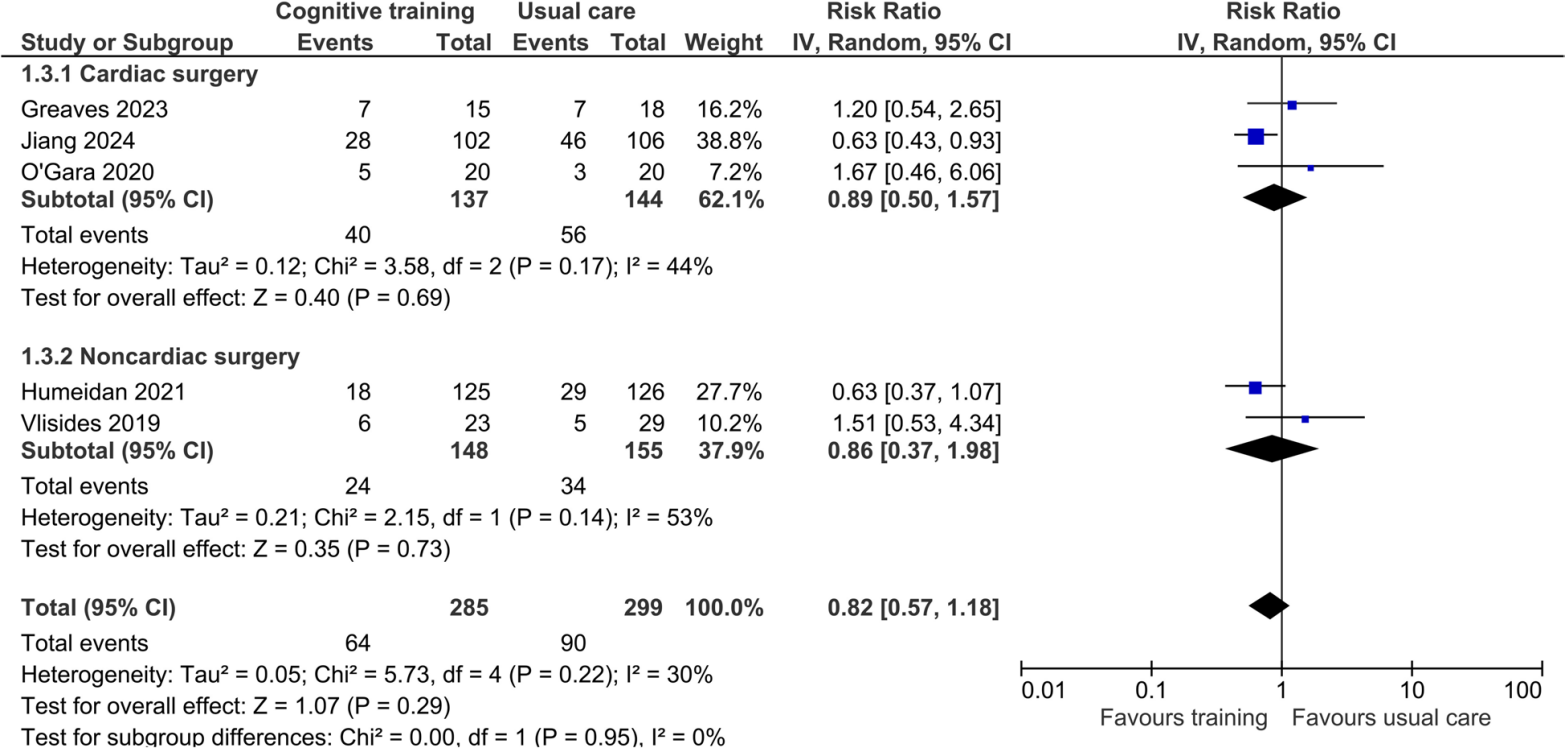
Forest plot of the effect of preoperative cognition training on the risk of postoperative delirium

Five trials (Greaves et al. 2023; Humeidan et al. 2021; Jiang et al. 2024; O'Gara et al. 2020; Vlisides et al. 2019) used the Confusion Assessment Methods (CAM, 3D-CAM (Marcantonio et al. 2014) and CAM-ICU) to assess the risk of POD, and two of these (Greaves et al. 2023; Humeidan et al. 2021) also used the Memorial Delirium Assessment Scale (Breitbart et al. 1997) and chart reviews to determine the occurrence of delirium during the weekends. Overall, the pooled effect of cognitive training from five trials (Greaves et al. 2023; Humeidan et al. 2021; Jiang et al. 2024; O'Gara et al. 2020; Vlisides et al. 2019) involving 584 participants did not reduce POD significantly compared to standard care (risk ratio [RR] 0.82, 95% confidence interval [CI] 0.57–1.18; *I*^2^ = 30%; Fig. 3). The certainty of this conclusion was, however, low due to the high risk of bias in some of the trials and imprecision of the overall effect. There were no subgroup differences in this result between cardiac and noncardiac surgical patients (*P* = 0.95). In the TSA, the cumulative *Z*-curves did not cross the trial sequential monitoring upper boundary or the futility boundaries and the estimated information size was 1217 (Online Resource 4).

### Postoperative cognitive dysfunction

**Fig. 4:**
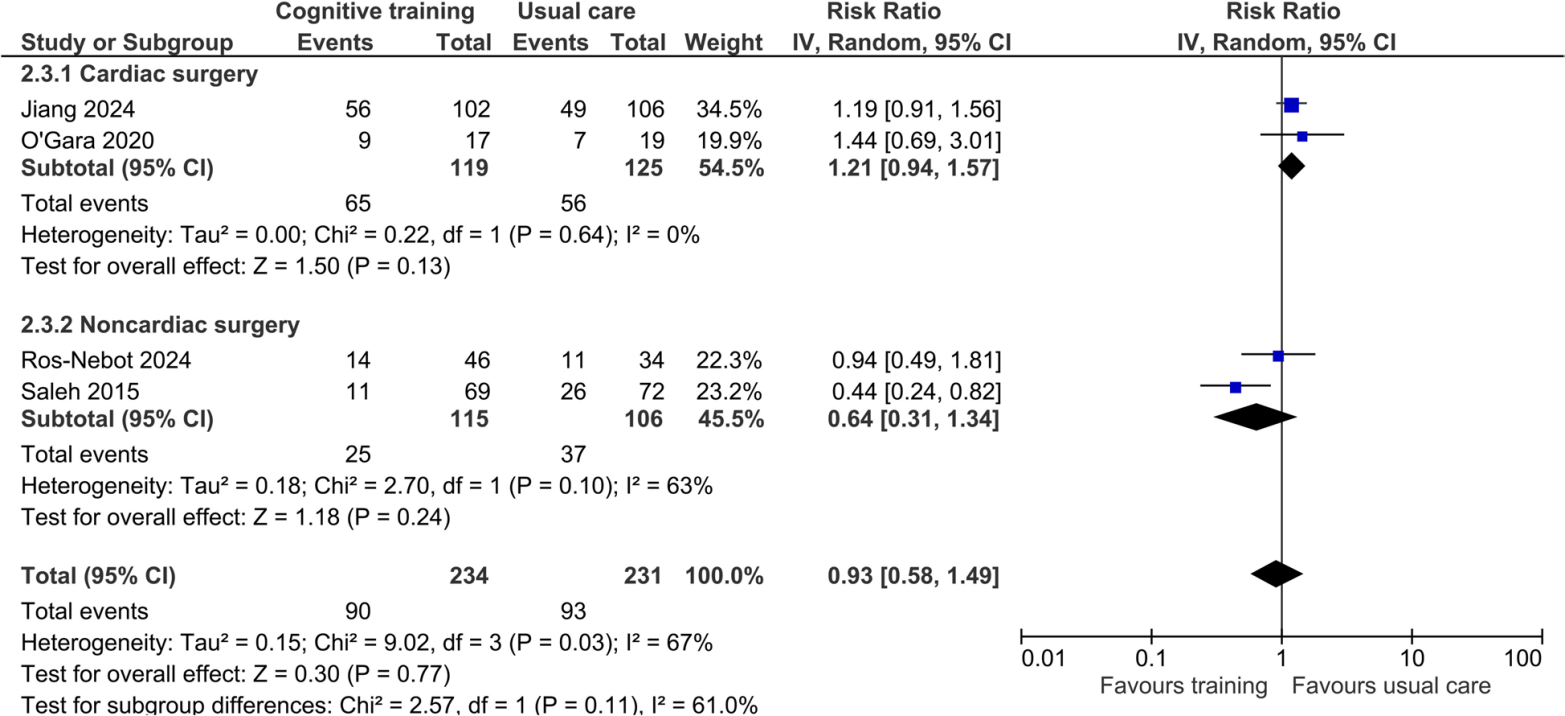
Forest plot of the effect of preoperative cognitive training on risk of early postoperative cognitive dysfunction

Four trials involving 465 participants used various methods to identify the risk of early POCD at hospital discharge or on postoperative day 7 (Jiang et al. 2024; O'Gara et al. 2020; Ros-Nebot et al. 2024; Saleh et al. 2015). Two trials (Jiang et al. 2024; O'Gara et al. 2020) defined POCD as one standard deviation (SD) decrease in MoCA score relative to the participant’s baseline. By contrast, Saleh and colleagues (Saleh et al. 2015) defined POCD as one SD decrease in two or more tests (out of eight) in a series of neuropsychological battery tests. Another study (Ros-Nebot et al. 2024) assessed POCD with three tests (Montejo Carrasco et al. 2012; Rami et al. 2010; Borson et al. 2003), and one of them—Mini-Cog test (Borson et al. 2003)—has previously been validated against MMSE for mild cognitive impairment (Li et al. 2018). Overall, the pooled effect of cognitive prehabilitation training did not reduce the risk of POCD but substantial heterogeneity existed (RR 0.93, 95% CI 0.58–1.49; *I*^2^ = 67%; Fig. 4). The certainty of this conclusion was very low due to high risk of bias in the pooled trials, heterogeneity, and the results were imprecise. There was no difference in the risk of POCD between cardiac and noncardiac surgical patients (*P* = 0.11). In the TSA, the cumulative *Z*-curves did not cross the trial sequential monitoring upper boundary or the futility boundaries and the estimated information sizes was 1126 (Online Resource 5).

As there were substantial methodological variations among trials in measuring cognitive changes between baseline and up to 6 months after surgery (Greaves et al. 2023; Jiang et al. 2024; O'Gara et al. 2020; Ros-Nebot et al. 2024; Vlisides et al. 2019), pooling the changes over time from baseline between trials together was deemed to be inappropriate. In brief, Ros-Nebot and colleagues (Ros-Nebot et al. 2024) reported that cognitive training significantly improved performance in the Memory Failures of Everyday Questionnaire (MFE) (Breitbart et al. 1997), the Memory Alteration Test (Montejo Carrasco et al. 2012), and the Mini-Cog (Rami et al. 2010) at 1 month after surgery. However, this finding was not confirmed by other trials. No significant changes between groups in psychomotor speed, visual memory, spatial working memory, executive function, and global cognition using the Addenbrooke’s Cognitive Examination (Hsieh et al. 2013) and the Cambridge Neuropsychological Test Automated Battery (Sahakian and Owen 1992) tests were found at discharge, 4 and 6 months after surgery (Greaves et al. 2023). Cognitive function 1 month after surgery as assessed using the modified telephone interview for cognitive status (TICS-M) (Cook et al. 2009) did not differ between groups (Jiang et al. 2024). In another trial, there were no differences between the groups for the median MoCA scores at discharge, 1, 3, and 6 months after surgery (O'Gara et al. 2020). Finally, there were no mean differences between groups in the three NIH Toolbox (Weintraub et al. 2013) cognitive tests on postoperative day 3 in another study (Vlisides et al. 2019).

### Compliance

The definition of compliance and adherence to the recommended hours of training varied between studies (Table 2). Two trials (Ros-Nebot et al. 2024; Saleh et al. 2015) adopted a per-protocol analysis and only included participants with a complete adherence to the prescribed program without reporting the compliance rate. The participants’ compliance rate was either not reported (Ros-Nebot et al. 2024; Saleh et al. 2015) or mostly below 70% (Greaves et al. 2023; Humeidan et al. 2021; O'Gara et al. 2020; Vlisides et al. 2019).

**Table 2:** Compliance with cognitive training in included trials

Author, year	Compliance definition	Recommended number of training hours	Actual median (IQR) training hours	Compliance rate
Greaves et al. 2023	Percentage of participants attending 3 or more preoperative sessions	2.6 to 3	Not reported	68%
Humeidan et al. 2021	a. Percentage of participants meeting recommended number of training hours	10	4.6 (1.3 to 7.4)	9%
	b. Completion of some exercises	Not reported	Not reported	97%
Jiang et al. 2024	Percentage of participants meeting 3-h training time	10	6.0 (5.0 to 7.0)	94%
O'Gara et al. 2020	a. Percentage of recommended number of training hours completed by the participants	≥ 5	4.1 (2.3 to 8.9)	39%
	b. Total preoperative training	Not reported	≥ 1 h	51%
Ros-Nebot et al. 2024	Not defined	2.5	Not reported	Not reported
Saleh et al. 2015	Not defined	3	Not reported	Not reported
Vlisides et al. 2019	Percentage of participants meeting recommended number of training hours	2.3	Not reported	17%

*IQR* Interquartile range

## Discussion

While there is no doubt that cognitive training, especially long-term, is good for our brain health (Mewborn et al. 2017; Rebok et al. 2014), its utility as a short-term prehabilitation program before surgery has not been established. Our current systematic review could not confirm the benefits of cognitive prehabilitation in reducing the risk of POD and POCD. It is noteworthy that the sample sizes of the pooled studies were underpowered as suggested by both TSA, patient compliance was not high and they had a substantial risk of bias. Specifically, there was a large discrepancy between the planned number of hours of training and the actual training achieved by most participants who were over 65 years old. These results have clinical and research relevance and require further consideration.

First, a recent systematic review showed that perioperative cognitive training reduced the risk of POCD (RR 0.50, 95% CI 0.28–0.89; *I*^2^ = 61%) but not for POD (RR 0.64; 95% CI 0.29–1.43; *I*^2^ = 67%) (Zhao et al. 2023). However, this review had pooled an observational study (Lee et al. 2013) with the RCTs and included trials that involved more than cognitive training alone (such as with rehabilitation exercise (Duan et al. 2022) or with early mobilization and nutritional assistance) (Chen et al. 2017). Thus, the benefits of short-term cognitive prehabilitation on postoperative neurological outcomes remain scientifically unproven.

Second, in another systematic review of 52 RCTs involving 4885 cognitively healthy older adults (Lampit et al. 2014), home-based computerized cognitive training (≥ 4 h) training did not appear to be effective (Hedges’ *g* = 0.09, 95% CI − 0.02 to 0.21; *P* = 0.11) compared to group-based training (Hedges’ *g* = 0.29, 95% CI 0.21–0.38; *P* < 0.001) on all verbal memory, nonverbal memory, working memory, processing speed, attention, visuospatial skills, and executive functions outcomes combined. The results of this review suggest that patient compliance is a key to any cognitive training. Consistent with this important point about any training programs, we observed no significant benefits in those studies that used home-based, unsupervised, computerized training studies (Greaves et al. 2023; Humeidan et al. 2021; Jiang et al. 2024; Ros-Nebot et al. 2024; Vlisides et al. 2019) in contrast to the positive effect of a supervised, hospital-based cognitive training (Saleh et al. 2015).

Thirdly, both POD and POCD TSA results suggest that the current evidence is insufficient to exclude the effectiveness of preoperative cognitive training interventions on postoperative neurological outcomes given the well-established benefits of long-term cognitive training on cognition in the elderly.

We acknowledge several limitations to our systematic review. First, publication bias may be present despite an extensive search, without language restrictions, in six electronic databases for eligible trials to be included in the systematic review. Due to the small numbers of trials (< 10), formal statistical tests for publication bias could not be undertaken (Higgins et al. 2023). Second, there was an insufficient number of included trials to perform a meta-regression analysis to explore the role of baseline cognitive status associated with cognitive training programs on the risk of POD and POCD. Short preoperative cognitive exercise training may provide different “benefit” in patients with or without mild cognitive impairments. Finally, we found the quality reporting of cognitive training interventions to be only fair to moderate. As such, a more detailed characterization of the intervention in any future trials will be pivotal to confirm reproducibility.

Recommendations for future trials to demonstrate cognitive gains in carefully selected patients while minimizing potential negative consequences such as training fatigue and increased preoperative anxiety levels (Vlisides et al. 2019) have been articulated by Vlisides and colleagues (Vlisides et al. 2020). Similarly, standardizing the reporting of “intervention compliance” will be helpful. The multiple definitions used to define compliance by various trials prevented meaningful inference to conclude about the optimal time, duration, and modality to conduct cognitive training before surgery.

## Conclusion

This systematic review showed that the strength of current evidence is insufficient to exclude the effectiveness of cognitive prehabilitation in reducing the risk of POD and POCD. A sample size of over 1000 patients with protocols to ensure high patient compliance will be essential in designing future RCTs in this important area of perioperative medicine.

## Supplementary Information

Additional file 1. Online Resource 1: Search Strategy. Online Resource 2: List of excluded studies. Online Resource 3: TIDieR checklist assessment. Online Resource 4: Trial sequential analysis for prevention of postoperative delirium. Online Resource 5: Trial sequential analysis for prevention of postoperative cognitive dysfunction.

## Data Availability

The authors confirm that the data supporting the findings of this study are available within the article and its supplementary materials.

## Abbreviations

CAM: Confusion Assessment Method
CAM-ICU: Confusion Assessment Method for the Intensive Care Unit
CCT: Computerized cognitive training
CENTRAL: Cochrane Central Register of Controlled Trials
GRADE: Grading of Recommendations, Assessment, Development and Evaluation
MFE: Memory Failures of Everyday Questionnaire
MMSE: Mini-Mental State Examination
MoCA: Montreal Cognitive Assessment
POCD: Postoperative cognitive dysfunction
POD: Postoperative delirium
PRISMA: Preferred Reporting Items for Systematic Reviews and Meta-Analyses
RCT: Randomized controlled trials
TICS-M: Modified Telephone Interview for Cognitive Status
TIDieR: Template for intervention description and replication
TSA: Trial sequential analysis
