# Supplementary material for: Preoperative cognitive training for the prevention of postoperative delirium and cognitive dysfunction: a systematic review and meta-analysis
Source: Perioper Med (Lond). 2024 Nov 30;13:113. doi: 10.1186/s13741-024-00471-y (PMC11607966; doi:10.1186/s13741-024-00471-y)
Supplement: Supplementary file 1 — Additional file 1. Online Resource 1: Search Strategy. Online Resource 2: List of excluded studies. Online Resource 3: TIDieR checklist assessment. Online Resource 4: Trial sequential analysis for prevention of postoperative delirium. Online Resource 5: Trial sequential analysis for prevention of postoperative cognitive dysfunction. [file 13741_2024_471_MOESM1_ESM.pdf]

Electronic Supplementary Material

Preoperative cognitive training for the prevention of postoperative delirium and cognitive dysfunction: a systematic review and meta-analysis

Online Resource 1: Search Strategy

Online Resource 2: List of excluded studies

Online Resource 3: TIDieR checklist assessment

Online Resource 4: Trial sequential analysis for prevention of postoperative delirium

Online Resource 5: Trial sequential analysis for prevention of postoperative cognitive dysfunction

Online Resource 1: Search Strategy

OVID MEDLINE

1	((cognitive OR cognition OR neurocognitive OR brain OR mental) adj2 (training OR exercise OR therapy OR game OR intervention OR stimulation OR activity)) OR CCT
2	delirium/
3	((postoperative OR postsurgical OR post-operative OR post-surgical) adj2 (delirium)) OR POD
4	((postoperative OR postsurgical OR post-operative OR post-surgical) adj2 (cognitive OR neurocognitive) adj2 (dysfunction OR decline OR impairment OR disorder)) OR POCD
5	Postoperative cognitive complications/
6	2 OR 3 OR 4 OR 5
7	1 AND 6
8	Randomized Controlled Trial.pt.
9	randomized controlled trial.pt.
10	controlled clinical trial.pt.
11	randomized.ab.
12	placebo.ab.
13	drug therapy.fs
14	randomly.ab.
15	trial.ab.
16	groups.ab.
17	8 or 9 or 10 or 11 or 12 or 13 or 14 or 15 or 16
18	exp animals/ not humans.sh.
19	17 not 18
20	7 and 19

OVID EMBASE

1	((cognitive OR cognition OR neurocognitive OR brain OR mental) adj2 (training OR exercise OR therapy OR game OR intervention OR stimulation OR activity)) OR CCT
2	delirium/
3	((postoperative OR postsurgical OR post-operative OR post-surgical) adj2 (delirium)) OR POD
4	((postoperative OR postsurgical OR post-operative OR post-surgical) adj2 (cognitive OR neurocognitive) adj2 (dysfunction OR decline OR impairment OR disorder)) OR POCD
5	Postoperative cognitive complications/
6	2 OR 3 OR 4 OR 5
7	1 AND 6
8	"randomized controlled trial (topic)"/
9	10 not ((exp animal/ or nonhuman/) not exp human/)
10	9 and 11

Web of Science

1	((cognitive OR cognition OR neurocognitive OR brain OR mental) NEAR/2 (training OR exercise OR therapy OR game OR intervention OR stimulation OR activity)) OR CCT
2	((postoperative OR postsurgical OR post-operative OR post-surgical) NEAR/2 (delirium)) OR POD
3	((postoperative OR postsurgical OR post-operative OR post-surgical) NEAR/3 (cognitive OR neurocognitive) NEAR/3 (dysfunction OR decline OR impairment OR disorder)) OR POCD
4	2 OR 3 (outcome)
5	1 AND 4
6	TS= clinical trial* OR TS=research design OR TS=comparative stud* OR TS=evaluation stud* OR TS=controlled trial* OR TS=follow-up stud* OR TS=prospective stud* OR TS=random* OR TS=placebo* OR TS=(single blind*) OR TS=(double blind*)
7	5 AND 6

Scopus

Query String	TITLE-ABS-KEY(((cognitive OR cognition OR neurocognitive OR brain OR mental) W/2 (training OR exercise OR therapy OR game OR intervention OR stimulation OR activity)) OR CCT)<br><br>AND<br><br>(TITLE-ABS-KEY(((postoperative OR postsurgical OR post-operative OR post-surgical) W/2 delirium) OR POD)<br><br>OR<br>TITLE-ABS-KEY(((postoperative OR postsurgical OR post-operative OR post-surgical) W/3 (cognitive OR neurocognitive) W/3 (dysfunction OR decline OR impairment OR disorder)) OR POCD))<br><br>AND<br><br>(INDEXTERMS ("clinical trials" OR "clinical trials as a topic" OR "randomized controlled trial" OR "Randomized Controlled Trials as Topic" OR "controlled clinical trial" OR "Controlled Clinical Trials" OR "random allocation" OR "Double-Blind Method" OR "Single-Blind Method" OR "Cross-Over Studies" OR "Placebos" OR "multicenter study" OR "double blind procedure" OR "single blind procedure" OR "crossover procedure" OR "clinical trial" OR "controlled study" OR "randomization" OR "placebo")) OR (TITLE-ABS-KEY (("clinical trials" OR "clinical trials as a topic" OR "randomized controlled trial" OR "Randomized Controlled Trials as Topic" OR "controlled clinical trial" OR "Controlled Clinical Trials as Topic" OR "random allocation" OR "randomly allocated" OR "allocated randomly" OR "Double-Blind Method" OR "Single-Blind Method" OR "Cross-Over Studies" OR "Placebos" OR "cross-over trial" OR "single blind" OR "double blind" OR "factorial design" OR "factorial trial"))) OR (TITLE-ABS (clinical trial* OR trial* OR rct* OR random* OR blind*))

CINAHL Complete

1	((cognitive OR cognition OR neurocognitive OR brain OR mental) N2 (training OR exercise OR therapy OR game OR intervention OR stimulation OR activity)) OR CCT
2	(MH "Cognitive Therapy") OR (MH "Rehabilitation, Cognitive") OR (MH "Cognition") OR (MH "Cognitive Stimulation (Iowa NIC)") OR (MH "Cognitive Therapy (Iowa NIC)") OR (MH “Cognitive Remediation”)
3	1 OR 2
4	((postoperative OR postsurgical OR post-operative OR post-surgical) N2 (delirium)) OR POD
5	((postoperative OR postsurgical OR post-operative OR post-surgical) N2 (cognitive OR neurocognitive) N2 (dysfunction OR decline OR impairment OR disorder)) OR POCD
6	(MH "Delirium") OR (MH "Cognition Disorders") OR (MH "Delirium Management (Iowa NIC)") OR (MH “Postoperative Complications”)

7	4 OR 5 OR 6
8	3 AND 7
9	8 (with human and RCT filter)

Cochrane Central Register

1	((cognitive OR cognition OR neurocognitive OR brain OR mental) NEAR/2 (training OR exercise OR therapy OR game OR intervention OR stimulation OR activity)) OR CCT
2	MeSH descriptor: [Delirium] explode all trees
3	((postoperative OR postsurgical OR post-operative OR post-surgical) NEAR/2 (delirium)) OR POD
4	MeSH descriptor: [Postoperative Cognitive Complications] explode all trees
5	((postoperative OR postsurgical OR post-operative OR post-surgical) NEAR/3 (cognitive OR neurocognitive) NEAR/3 (dysfunction OR decline OR impairment OR disorder)) OR POCD
6	2 OR 3 OR 4 OR 5 (outcome)
7	1 AND 6
8	7 (with Trial filter)

Online Resource 2: List of excluded studies

		Reason
1	Butz M, El Shazly J, Sammer G, Tschernatsch M, Kastaun S, Yenigün M, Braun T, Kaps M, Böning A, Puvogel U, Bachmann G, Mengden T, Schönburg M, Gerriets T, Juenemann M. Decreasing postoperative cognitive deficits after heart surgery: protocol for a randomized controlled trial on cognitive training. Trials. 2019;20(1):733. doi: 10.1186/s13063-019-3799-0.	Wrong timing of cognitive training intervention (postop)
2	Duan S, Liao Y, Tang Y, Zhang B, Peng M, Tong J, Ouyang W, LE Y. Short-term perioperative cognitive therapy combined with rehabilitation exercise reduces the incidence of neurocognitive disorder in elderly patients: a randomized controlled trial. Minerva Anestesiol. 2022;88(3):145-155. doi: 10.23736/S0375-9393.21.15877-8.	Cognitive training cannot be isolated
3	Fahimi K, Abbasi A, Zahedi M, Amanpour F, Ebrahimi H. The effects of multimedia education on postoperative delirium in patients undergoing coronary artery bypass graft: A randomized clinical trial. Nurs Crit Care. 2020;25(6):346-352. doi: 10.1111/nicc.12473.	Wrong timing of cognitive training intervention (postop)
4	Kulason K, Nouchi R, Hoshikawa Y, Noda M, Okada Y, Kawashima R. The Beneficial Effects of Cognitive Training With Simple Calculation and Reading Aloud (SCRA) in the Elderly Postoperative Population: A Pilot Randomized Controlled Trial. Front Aging Neurosci. 2018;10:68. doi: 10.3389/fnagi.2018.00068.	Wrong timing of cognitive training intervention (postop)
5	Kulason K, Nouchi R, Hoshikawa Y, Noda M, Okada Y, Kawashima R. The beneficial effects of cognitive training with simple calculation and reading aloud in an elderly postsurgical population: study protocol for a randomized controlled trial. Trials. 2016;17:334. doi: 10.1186/s13063-016-1476-0.	Wrong timing of cognitive training intervention (postop)
6	O'Gara B, Marcantonio ER, Pascual-Leone A, Shaefi S, Mueller A, Banner-Goodspeed V, Talmor D, Subramaniam B. Prevention of Early Postoperative Decline (PEaPoD): protocol for a randomized, controlled feasibility trial. Trials. 2018 Dec 11;19(1):676. doi: 10.1186/s13063-018-3063-z.	Wrong timing of cognitive training intervention (postop)
7	Rengel KF, Mehdiratta N, Vanston SW, Archer KR, Jackson JC, Thompson JL, Pandharipande PP, Hughes CG. A randomised pilot trial of combined cognitive and physical exercise prehabilitation to improve outcomes in surgical patients. Br J Anaesth. 2021;126(2):e55-e57. doi: 10.1016/j.bja.2020.11.004.	Cognitive training cannot be isolated
8	Shi Z, Song J, Chang H, Zhang Y. Effects of preoperative psychological intervention on early postoperative cognitive dysfunction after off-pump coronary artery bypass surgery. Biomed Res. 2017;28(7):2909-12.	Wrong non-cognitive training intervention
9	Song Y, Cui X, Zhang Y, Gao H, Cai Q, Mu Z. Home-Based Computerized Cognitive Training for Postoperative	Wrong timing of cognitive

	Cognitive Dysfunction After Lung Transplantation in Elderly Population: A Randomized Controlled Trial. J Nerv Ment Dis. 2019;207(8):693-699. doi: 10.1097/NMD.0000000000001032.	training intervention (postop)
10	Tarasova I.V., Trubnikova O.A., Kuhareva I.N., Sosnina A.S., Kupriyanova D.S., Shesternin V.G., Nagirnyak O.A., Barbarash O.L. Effects of dual-task rehabilitative training in the early postoperative period after direct myocardial revascularization. Complex Issues of Cardiovascular Diseases. 2021;10(3):15-25. (In Russ.) https://doi.org/10.17802/2306-1278-2021-10-3-15-25.	Wrong timing of cognitive training intervention (postop)
11	Wang YY, Yue JR, Xie DM, Carter P, Li QL, Gartaganis SL, Chen J, Inouye SK. Effect of the Tailored, Family-Involved Hospital Elder Life Program on Postoperative Delirium and Function in Older Adults: A Randomized Clinical Trial. JAMA Intern Med. 2020;180(1):17-25. doi: 10.1001/jamainternmed.2019.4446.	Wrong non-cognitive training intervention
12	ACTRN12617000513314: Cognitive and Lifestyle interventions to Understand the impact on the Elderly of anaesthesia and Surgery (The CLUES trial) https://anzctr.org.au/Trial/Registration/TrialReview.aspx?ACTRN=12617000513314.	Withdrawn protocol due to lack of funding/ staff/facilities
13	NCT03094988: COgnitive and Physical Exercise (COPE) Prehabilitation Pilot Feasibility Study https://classic.clinicaltrials.gov/ct2/show/NCT03094988.	Cognitive training cannot be isolated
14	NCT02747784: Evaluation to Assess Cognitive Training for the Prevention of Post-operative Cognitive Decline https://clinicaltrials.gov/study/NCT02747784.	Study terminated
15	ACTRN12619001778178: Perioperative Enhancement of Cognitive Trajectory (The PROTECT trial) https://anzctr.org.au/Trial/Registration/TrialReview.aspx?ACTRN=12619001778178.	Cognitive training cannot be isolated
16	NCT03353987: Preoperative Cognitive Training for Postoperative Cognitive Dysfunction (CogniTrain) https://classic.clinicaltrials.gov/ct2/show/NCT03353987.	Study terminated
17	NCT05143580: Preoperative Intervention to Reduce Delirium After Cardiac Surgery https://classic.clinicaltrials.gov/ct2/show/NCT05143580.	Wrong non-cognitive training intervention
18	ChiCTR-IOR-16009464: The effect of preoperative psychological intervention on postoperative cognitive dysfunction and NSE correlation analysis in elderly patients with abdominal operation [Internet]. https://www.chictr.org.cn/showprojEN.html?proj=16209.	Study terminated
19	NCT05523258: The Effect and Mechanism of Computerized Cognition training on the Incidence of Postoperative Cognitive Dysfunction After Cardiac Surgery https://classic.clinicaltrials.gov/ct2/show/NCT05523258	Wrong timing of cognitive training intervention (postop)

Online Resource 3: TIDieR checklist assessment

Yes: adequately reported
No: inadequately reported
N/A: item not applicable

Greaves et al. 2023

Item	Intervention	Control	Score
Brief name	Yes	N/A	2
Why	Yes	N/A	2
What materials	No	N/A	0
What procedures	Yes	N/A	2
Who provided	No	N/A	0
How	Yes	N/A	2
Where	No	N/A	0
When and how much	Yes	N/A	2
How well (planned)	Yes	N/A	2
How well (actual)	Yes	N/A	2
			**Total 14/20**

Humeidan et al. 2021

Item	Intervention	Control	Score
Brief name	Yes	N/A	2
Why	Yes	N/A	2
What materials	No	N/A	0
What procedures	Yes	N/A	2
Who provided	No	N/A	0
How	Yes	N/A	2
Where	No	N/A	0
When and how much	Yes	N/A	2
How well (planned)	No	N/A	0
How well (actual)	Yes	N/A	2
			**Total 12/20**

Jiang et al. 2024

Item	Intervention	Control	Score
Brief name	Yes	N/A	2
Why	Yes	N/A	2
What materials	Yes	N/A	0
What procedures	Yes	N/A	2
Who provided	No	N/A	0
How	Yes	N/A	2
Where	No	N/A	0
When and how much	Yes	N/A	2
How well (planned)	Yes	N/A	2
How well (actual)	Yes	N/A	2
			**Total 16/20**

O'Gara et al. 2020

Item	Intervention	Control	Score
Brief name	Yes	N/A	2
Why	Yes	N/A	2
What materials	No	N/A	0
What procedures	Yes	N/A	2
Who provided	No	N/A	0
How	Yes	N/A	2
Where	No	N/A	0
When and how much	Yes	N/A	2
How well (planned)	Yes	N/A	2
How well (actual)	Yes	N/A	2
			**Total 14/20**

Ros-Nebot et al. 2024

Item	Intervention	Control	Score
Brief name	Yes	N/A	2
Why	Yes	N/A	2
What materials	Yes	N/A	2
What procedures	Yes	N/A	2
Who provided	No	N/A	0
How	Yes	N/A	2
Where	No	N/A	0
When and how much	Yes	N/A	2
How well (planned)	Yes	N/A	2
How well (actual)	No	N/A	0
			**Total 14/20**

Saleh et al. 2015

Item	Intervention	Control	Score
Brief name	Yes	N/A	2
Why	Yes	N/A	2
What materials	Yes	N/A	2
What procedures	Yes	N/A	2
Who provided	No	N/A	0
How	No	N/A	0
Where	Yes	N/A	2
When and how much	Yes	N/A	2
How well (planned)	No	N/A	0
How well (actual)	No	N/A	0
			**Total 12/20**

Vlisides et al. 2019

Item	Intervention	Control	Score
Brief name	Yes	N/A	2
Why	Yes	N/A	2
What materials	Yes	N/A	2
What procedures	No	N/A	0
Who provided	No	N/A	0
How	Yes	N/A	2
Where	Yes	N/A	2
When and how much	Yes	N/A	2
How well (planned)	No	N/A	0
How well (actual)	Yes	N/A	2
			**Total 14/20**

Online Resource 4: Trial sequential analysis for prevention of postoperative delirium

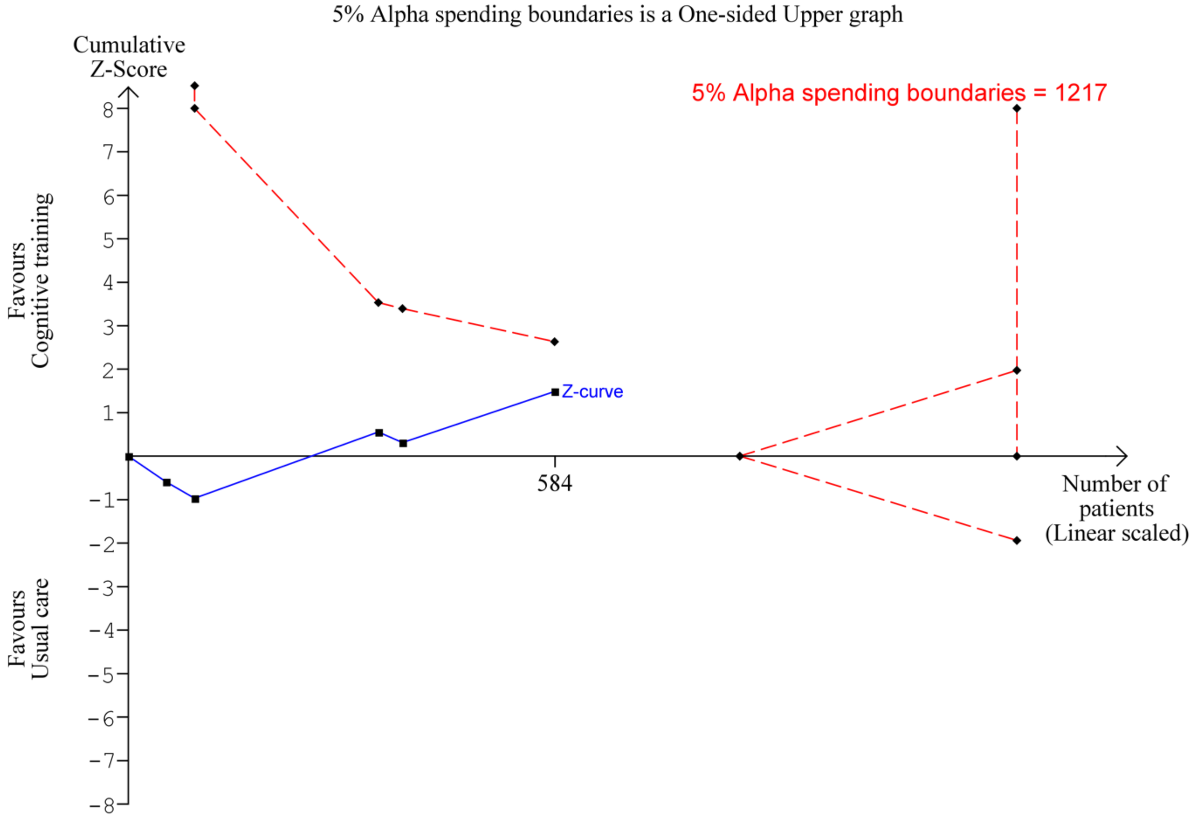

The red, inward-sloping, dashed lines (left) represent the trial sequential monitoring boundaries. To the right, the red outward sloping dashed lines (right) make up the futility region. The solid blue line is the cumulative Z-curve.

Online Resource 5: Trial sequential analysis for prevention of postoperative cognitive dysfunction

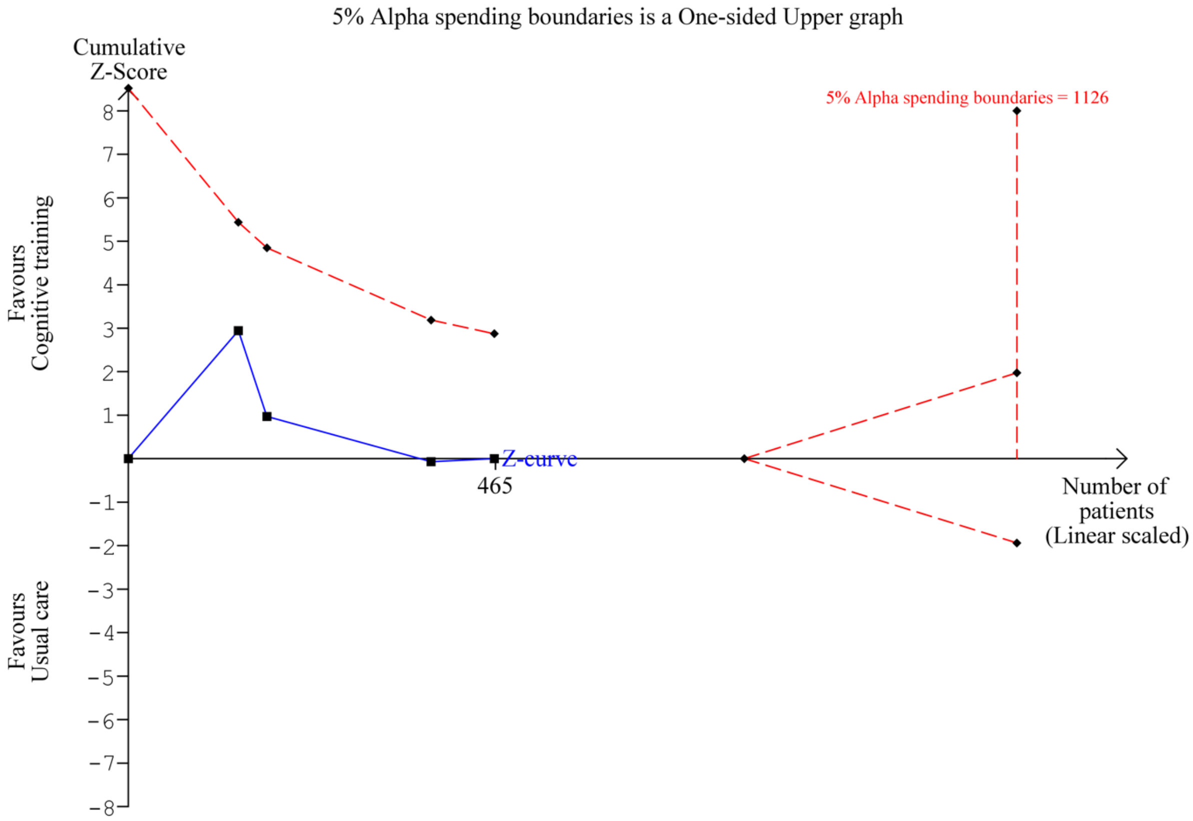

The red, inward-sloping, dashed lines (left) represent the trial sequential monitoring boundaries. To the right, the red outward sloping dashed lines (right) make up the futility region. The solid blue line is the cumulative Z-curve.
